# Regulatory role of human fibrocartilage stem cells in condyle osteochondroma

**DOI:** 10.1111/cpr.13342

**Published:** 2022-09-26

**Authors:** Qing Yin, Ruiye Bi, Haohan Li, Qianli Li, Peiran Li, Ruiyu Wang, Songsong Zhu

**Affiliations:** ^1^ State Key Laboratory of Oral Diseases & National Clinical Research Center for Oral Diseases, West China Hospital of Stomatology Sichuan University Chengdu China

## Abstract

**Objective:**

Osteochondroma is a common benign skeletal disorder for which different molecular and histological features of long bones have been reported. We investigated cell‐of‐origin and molecular mechanisms of a rare condylar osteochondroma (CO).

**Methods:**

Human fibrocartilage stem cells (hFCSCs) isolated from CO and normal condyle tissue were used for RNA sequencing, real‐time PCR, Western Blotting, immunohistology, flowcytometry, as well as for chondrogenic differentiation, proliferation, and apoptosis detection assays.

**Results:**

HFCSCs were fewer in number with weaker proliferative capacity and higher apoptosis ratio in the CO group. During the chondrogenic inducing process, hFCSCs from CO were prone to form more mature and hypertrophic cartilage. The result of RNA sequencing of hFCSCs from CO and normal condyle revealed a correlation between the PI3K/AKT signalling pathway and CO. Activated PI3K/AKT signalling might lead to functional changes in hFCSCs by enhancing cell apoptosis in the developmental process of CO. Increased expression of BCL2‐like protein 11 (BIM) in CO tissue also supports this conclusion. Furthermore, the activation of the PI3K/AKT pathway in TMJ of mice induced histological disorder and increased apoptosis in condylar cartilage.

**Conclusion:**

We conclude that the activation of PI3K/AKT signalling in hFCSCs of CO suggests a new hypothesis for the cell‐of‐origin of human CO and another possible target to treat it.

## Graphical abstract

This study reports that human fibrocartilage stem cells are the potential cell‐of‐origin of condylar osteochondroma. Activated PI3K/AKT signalling pathway regulates cell apoptosis of hFCSCs and further affects stem cell function, which leads to cartilage pathological disorder and hypertrophy.

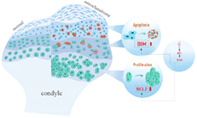

## 1 INTRODUCTION

Osteochondroma appears as a cartilage‐capped bony neoplasm formed by endochondral ossification.^1^ Osteochondroma can occur with multiple lesions, termed hereditary multiple exostoses (HME), but can also be found as a solidary lesion. Osteochondroma occurs less frequently in flat bones, especially craniofacial bone, than in the metaphyseal region of long bones. This low incidence relates to the intramembranous origin of most craniofacial bones.^2^, ^3^ Among craniofacial bones, the site of occasional tumorigenesis is the unilateral condyle or coronoid process. The excessive growth of condylar osteochondroma (CO) manifests as facial asymmetry, malocclusion, and even hearing symptoms.^4^, ^5^, ^6^, ^7^ It greatly affects facial aesthetics, masticatory function, and psychological health. Various theories have been proposed to explain the pathogenesis of osteochondroma. The cellular origin of osteochondroma appears to be ectopic cartilage formation in metaphyseal periosteum,^8^ growth plate chondrocytes^9^, ^10^ or multipotent mesenchymal cells in the densely‐packed region of the perichondral groove.^11^, ^12^ However, there is little information regarding the pathogenesis or cell‐of‐origin for CO.

A fibrocartilage stem cell (FCSCs) population has been found to reside in the condylar cartilage superficial zone according to animal studies.^13^ It plays an important role in condylar development, maturation, and maintenance of the temporomandibular joint (TMJ) microenvironment. Moreover, it participates in condylar subchondral vascularized bone formation.^14^ FCSCs could also be isolated from the superficial zone of the human condyle according to our previous study.^15^ Because of their stem‐cell‐like potencies and multiple regulatory functions, FCSCs are presumed to be one of the cell‐origins of CO.

In this study, we isolated FCSCs from human CO tissue for the first time and compared its characteristics with FCSCs from normal condyle. We investigated cell properties in osteochondromas in great detail and specifically searched for which signalling pathway regulates the function of FCSCs under pathological conditions. The data provided important evidence of FCSCs' relationship with the occurrence and development of CO.

## 2 MATERIALS AND METHODS

### 2.1 Primary cell isolation and culture

Human FCSCs (hFCSCs) were incubated in DMEM/High‐glucose medium (SH30243, Hyclone, USA) supplemented with 20% FBS (10099‐141‐FBS, Gibco, USA) and 1% Penicillin/Streptomycin (15140122, Gibco, USA). Normal hFCSCs were isolated from the condylar pieces that were not able to internal fixed in patients with condylar‐comminuted fracture. The hFCSCs from osteochondroma were cultivated from patients with CO according with surgical indications. The superficial zone of condyles was dissected by intraocular microforcep, cut to 1 × 1 mm^2^ size pieces. After washing with PBS, all tissue masses were incubated in type I collagenase (2 mg/mL) at 37°C for 2 hours. Subsequently, the digested cell mass was gently resuspended in 4‐5 ml complete DMEM medium and incubated in a T25 culture flask at 37°C and 5% CO_2_ overnight to allow cell attachment.

All experiments involving human tissue were approved by IRB human Subjects of West China Hospital of Stomatology (Code Number WCHSIRB‐D‐2017‐010). The procedures followed with human condyle samples were in accordance with ethical standards by responsible committee for human experimentation and with the Helsinki Declaration. The source of all samples is recorded in Table S3.

### 2.2 MRNA library construction and sequencing

Total RNA was extracted using Trizol reagent following the manufacturer's protocol. The total RNA quantity and purity were analysed by Bioanalyzer 2100 and RNA 6000 Nano LabChip Kit (Agilent, CA, USA) with RNA integrity number (RIN) number >7.0. Approximately 10 ug of total RNA was subjected to isolate Poly (A) mRNA with poly‐T oligo attached magnetic beads (Invitrogen). Following purification, the mRNA is fragmented into small pieces using divalent cations under elevated temperature. Then the cleaved RNA fragments were reverse‐transcribed to create the final cDNA library in accordance with the protocol for the mRNA Seq sample preparation kit (Illumina, San Diego, USA). The average insert size for the paired‐end libraries was 300 bp (±50 bp). We then performed the paired‐end sequencing on an Illumina X10 sequence platform.

### 2.3 Ethical approval information

All animal experiments were performed under the licence (No. WCHSIRB‐D‐2020‐142) and protocol approved by Ethics Committee of West China Hospital of Stomatology and conform to ARRIVE guidelines. All animals were kept and bred in SPF‐class housing. Qing Yin, Ruiye Bi, and Songsong Zhu were aware of the group allocation at the different stages of the experiment.

### 2.4 Local injection in TMJ in mice

30 μM 740Y‐P (HY‐P0175, MedChemExpress) was added for 24 h in medium culturing rat FSCs. Ten 2‐week C57/B6 mice from same brood were divided randomly into two groups. One group was injected with activator and the other with normal saline. 30 μM 740Y‐P was injected to unilateral TMJ and, as control, normal saline was injected to the same side in NS group. After injection every 2 days for 4 weeks, we collected all the samples and performed H&E, Safranin O, and terminal deoxynucleotidyl transferase dUTP nick end labeling (TUNEL) staining.

Seven points was set from anterior to posterior part on mouse condyle's section (*n* = 8) to separate it averagely, then the thickness of hypertrophic zone corresponding to each point was measured and recorded on the line chart to show hypertrophic cartilage distribution.

### 2.5 Statistical analysis

All experiments were repeated at least three times. Student's t test and Wilcoxon's rank–sum test were used for comparisons between hFCSCs of normal condyle and osteochondroma. Multiple comparisons in western blot were evaluated by one‐way analysis of variance analysis with Tukey's test. The analyses were performed using IBM SPSS statistics 22 software and GraphPad Prism 6.0. Data are presented as means ± SD. *p* < 0.05 was considered statistically significant.

*Fluorescence‐activated cell sorting, EdU labelling, TUNEL assay, chondrogenic inducing differentiation, immunocytochemistry, immunohistochemistry staining, quantitative PCR, and western blot are showed in supplemental appendix*.

## 3 RESULTS

### 3.1 CO shows more hypertrophic and mature cartilage cap and abnormal subchondral bone structure

**FIGURE 1:**
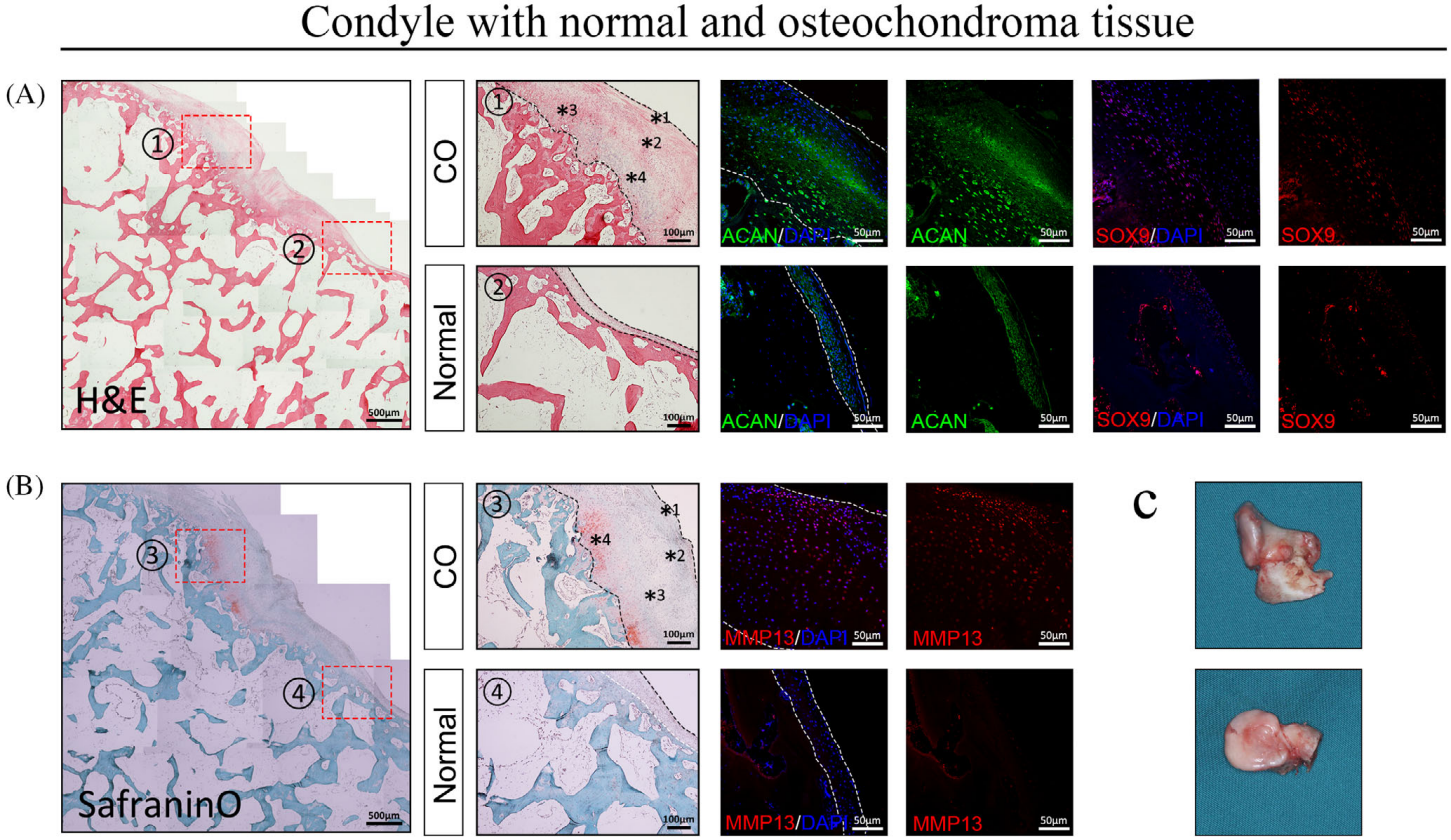
The histological characteristics of normal condyle and CO. (A),(B) Haematoxylin and eosin (H&E) staining, safranin O/fast green staining and immunofluorescence staining of one sample that contains both normal (2 and 4) and osteochondroma tissue (1 and 3). The osteochondroma tissue has a more abundant expression of ACAN, SOX9 and MMP13 compared with normal tissue. (C) Photograph showing protruded and hypertrophic appearance of the condyle. *1, superficial zone; *2, proliferating zone; *3, pre‐hypertrophic zone; *4, hypertrophic zone.

Pedunculated and sessile types of COs could both be observed clinically. We collected three special samples which included tumorous and normal parts of the same condyle (Figure 1C). A fibrous perichondrium covered the cartilage cap of the tumour and was continuous with the periosteum of the subchondral bone. The morphology of cartilage cap and the arrangement of chondrocytes were irregular in COs by both visual and pathological observation. H&E (Figure 1A, left) and Safranin O/Fast Green staining (Figure 1B, left) revealed that the tumorous part has a thicker fibrous superficial zone and more abundant cartilage matrix at the hypertrophic zone. Meanwhile, we also observed several abnormal cartilage islands in the subchondral bone.

Histological analyses were carried out to investigate how markers of cartilage differentiation changed in CO. Stronger expressions of SOX9, MMP13, and ACAN were observed at the superficial, flattened, and hypertrophic zones of CO samples (Figure 1A,B, right). These findings suggest that CO's histological structure and chondrogenic protein level differs from normal condyle.

### 3.2 HFCSCs from osteochondroma exhibit distinct stem cell features from hFCSCs in normal TMJ cartilage

The superficial zone was dissected from normal condyles and osteochondromas and used for the primary culture of human FCSCs (hFCSCs). We enriched hFCSCs using four mesenchymal stem cell surface markers and fluorescent activated cell sorting (FACS). HFCSCs were simultaneously incubated with CD34/CD73/CD90/CD105 primary antibodies and conjugated fluorescein and subjected to FACS. Using CD34‐/CD73+/CD90+/CD105+ as a standard, there was a lower proportion of hFCSCs in the mixed cell population from CO than from the normal sample (1.51 ± 0.97% versus 3.89 ± 1.12%) (Figure S1).

**FIGURE 2:**
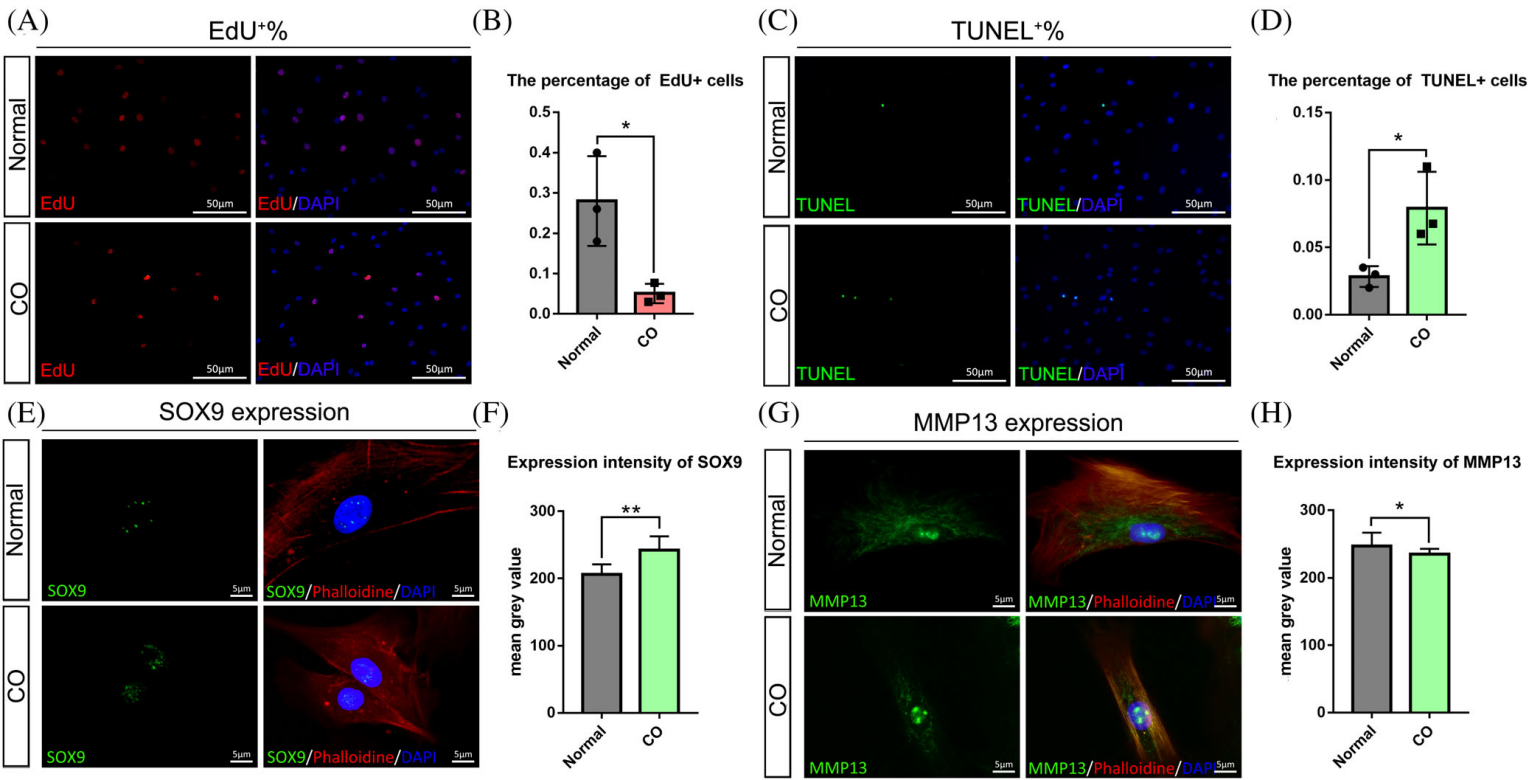
HFCSCs from CO have less proliferative capacity, a higher apoptotic percentage and different expression levels of SOX9 and MMP13 than normal. (A) hFCSCs were incubated with EdU (10 μM) for two hours followed by fixation. (C) hFCSCs were detected using TUNEL labeling and hFCSCs from CO showed more cell apoptosis. (B),(D) Semiquantitative analysis of the percentage of EdU/TUNEL positive cells. Immunofluorescent imaging was used to detect Alexa Fluor® 555‐stained‐EdU (red), TUNEL conjugated to FITC (green), and nuclei stained with DAPI (blue) at 10 × 20 (=200) magnification. (E),(G) SOX9 and MMP13 expression in hFCSCs by immunocytochemistry. HFCSCs grown on coverslips and fixed in 4% paraformaldehyde, blocked in 10% goat serum and incubated with anti‐SOX9 or MMP13 antibody. SOX9 or MMP13 was detected with anti‐rabbit IgG antibody conjugated with Alexa Fluor 488 (green fluorescence). (F),(H) The mean value of expression level was measured by mean grey value of 8‐bit images. Data were obtained from at least three samples per group and are expressed as the means ± S.D. of the percentage of total cells (*p ˂ 0.05, ***p ˂ 0.001).

We characterized the stem cell properties of hFCSCs from the tumorous environment by performing EdU labelling and TUNEL assay. Compared with normal hFCSCs, hFCSCs from CO showed slower cell propagation and a slightly higher cell apoptosis rate: 28 ± 6.4% versus 5.1 ± 1.4%, *p* < 0.05 and 2.8 ± 0.4% versus 7.9 ± 1.5%, *p* < 0.05 (Figure 2A–D). Thus, CO has a smaller hFCSCs population with different proliferative and apoptotic characteristics compared with a normal condyle.

To confirm the chondrogenic differentiation potential of hFCSCs, we first measured the expression intensity of SOX9 and MMP13 as chondrogenic differentiation markers of the two types of hFCSCs using cell slides and immunocytochemistry. Expression of the early marker SOX9 in hFCSCs from CO was 1.2‐fold higher than in normal hFCSCs (Figure 2E,F). Meanwhile, the expression of the hypertrophic marker MMP13 in hFCSCs from CO was only 0.9‐fold of normal (Figure 2G,H).

**FIGURE 3:**
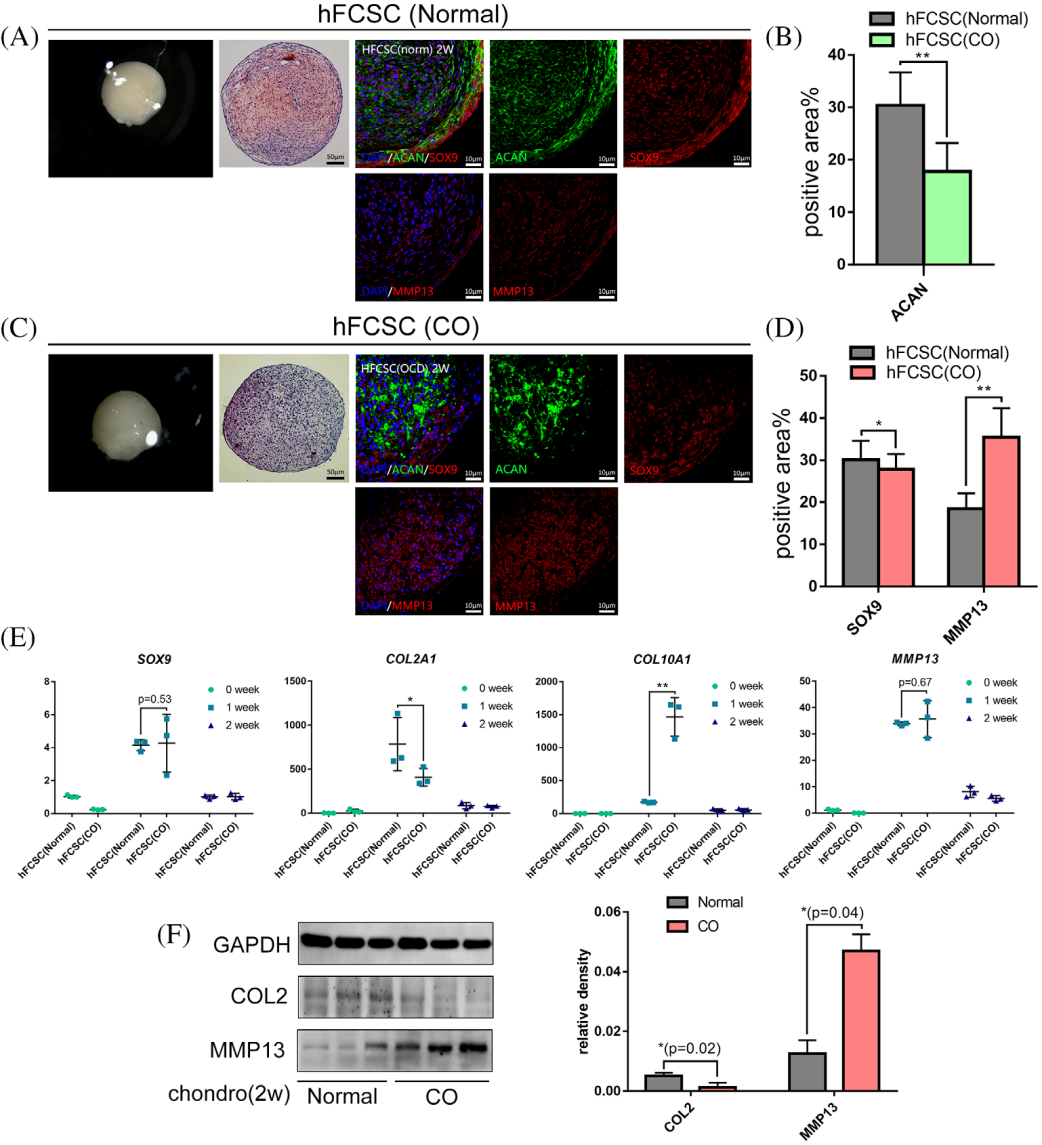
HFCSCs from CO exhibit different pattern during chondrogenic induction process. Pellets of hFCSCs were incubated for one and two weeks in chondrogenic medium. (A),(B) Representative images of photos taken by Stereo Microscope (left), safranin O/fast green staining (center) and ACAN, SOX9 and MMP13 (right) at the second week. (B),(D) The comparation of expression level was calculated by fluorescent area proportion. (E) Relative mRNA levels of *SOX9*, *COL2A1*, *COL10A1* and *MMP13* in induced pellets were measured by qRT‐PCR. Gene expression at each stage is given relative to the level on day 0; *n* = 4. (F) Expression of COL2 and MMP13 protein in pellets incubated in chondrogenic medium for two weeks, as determined by Western blot. The graph at right shows the relative density of measured proteins, GAPDH was used as a reference. Data are presented as mean ± S.D. Statistical analysis was performed using Wilcoxon's rank‐sum test (*p ˂ 0.05, **p ˂ 0.01).

We then confirmed the gene expression patterns of SOX9, ACAN, COL2, COL10, and MMP13 during chondrogenic differentiation. Gene expression and protein level showed different patterns. Expression of chondrogenic related genes increased in the first week but fell during the second week. The gene expression of COL2A1 in CO‐hFCSC inducing pellets was significantly weaker than normal (0.5‐fold, *p* < 0.05). The gene expression of COL10A1 in CO pellets was significantly higher than normal (10‐fold, *p* < 0.01). MMP13 and SOX9 did not exhibit significant differences between the two groups at the genetic level (Figure 3E). Subsequently, protein expression was determined by immunohistochemistry and Western Blot. Safranin O/Fast Green staining showed that more proteoglycans formed in the normal group (Figure 3A,C, left). The protein expression of chondrogenic differentiation markers like SOX9 and ACAN in CO pellets was weaker than in normal pellets (Figure 3B,D). The percentage of positive area were: SOX9 (27.9 ± 3.6% versus 30.1 ± 4.5%, *p* < 0.05), ACAN (17.8 ± 5.4% versus 30.4 ± 6.3%, *p* < 0.01) (Figure 3B,D). The expression of the hypertrophic differentiation marker MMP13 in CO pellets was stronger than in normal pellets, 35.4 ± 6.9% versus 18.5 ± 3.6%, *p* < 0.01 (Figure 3D). The lower expression of COL2 and stronger expression of MMP13 in Western Blot result were consistent with immunostaining result (Figure 3F). Together these data indicate that hFCSCs from CO exhibit a specific chondrogenic differentiation capacity and are prone to entering the later stage of the chondrogenesis inducing process.

### 3.3 Several signalling pathways regulate the function of hFCSCs during pathological process of CO

We next investigated the possible functional molecular mechanisms of hFCSCs in the pathological processes of CO. We performed a transcriptomic analysis of two types of hFCSCs by RNA‐sequencing and compared the gene expression profiles between those two groups. The hFCSCs from three CO samples and four normal samples were isolated by FACS and then tested.

**FIGURE 4:**
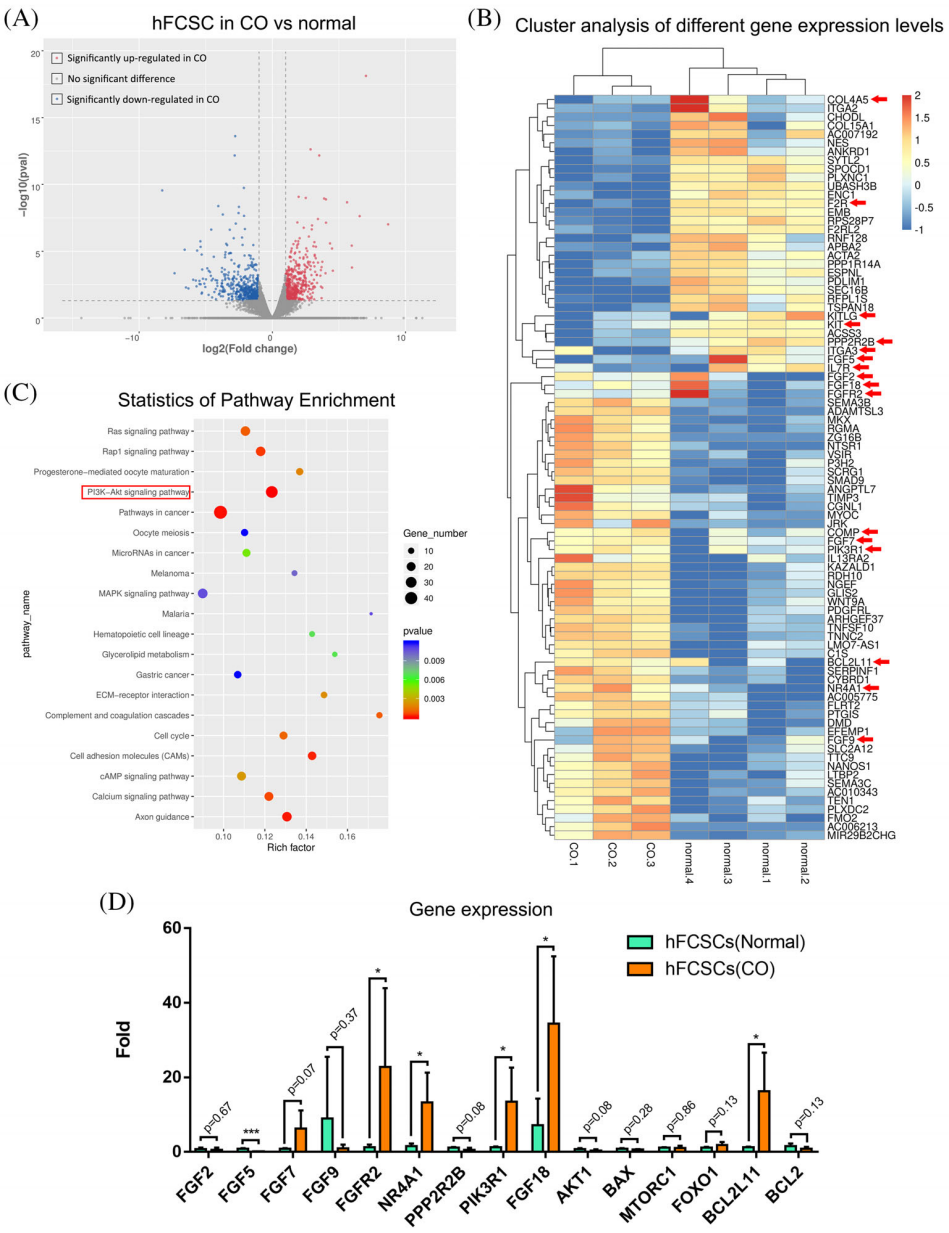
HFCSCs from CO show altered expression of several genes in the PI3K/AKT signalling pathway. (A) RNA‐seq analysis was performed with hFCSCs residing in CO and normal condyle. Volcano diagram shows that the expression of 499 up‐regulated genes and 525 down‐regulated genes. (B) 89 genes selected from the 1024 differential genes identified by RNA‐Seq analysis which were related to several cell processes, such as chondrogenic differentiation, cell proliferation and others. A heat map of these genes was generated. Genes in PI3K/AKT signalling pathway are highlighted with red arrows. (C) The related signalling pathways were analysed based on KEGG database and PI3K/AKT signalling was selected after considering the number of genes, p‐value and rich factor. (D) Some genes in PI3K/AKT signalling were significantly upregulated or downregulated as detected by qRT‐PCR (*p ˂ 0.05, ***p ˂ 0.001).

The Volcano plot (Figure 4A) shows a striking lack of similarity in the gene expression patterns between CO and normal samples. Using a t‐test, we identified 1024 genes with significantly different expression between two groups (false discovery rate [FDR] <0.05), including 499 up‐regulated genes and 525 down‐regulated genes in the CO group compared to the normal group.

Based on RNA‐seq data, we performed a functional signalling pathway analysis of genes differentially expressed between two groups using the Kyoto Encyclopedia of Genes and Genomes (KEGG). Functional pathways that significantly differ between CO and normal hFCSCs are depicted in Figure 4B. Synthesizing rich factor, p‐value, and gene number data, the PI3K/AKT signalling pathway appears to be one of the most significant affected pathways. All related genes of PI3K/AKT signalling pathways are listed in Table S1. The heat map in Figure 4C shows that the CO group had higher expression of some genes related to cartilage development, collagen fibril organization, chondrocyte development, and endochondral bone growth. Importantly, the data show higher expression for several genes closely related to PI3K/AKT signalling (Figure 4C, red arrow). Several mRNAs related to stem cell differentiation were significantly down regulated. Among those, many of the genes are related to the PI3K/AKT signalling pathway and show a significant difference in expression.

We verified the results of the RNA‐seq analysis by validating the levels of 14 hub genes (*FGF2, FGF5, FGF7, FGF9, FGF18, FGFR2, NR4A1, PPP2R2B, PIK3R1, AKT1, BAX, MTORC1, FOXO1, BCL2L11, BCL2*) in the PI3K/AKT signalling pathway by real‐time PCR. The expression levels of these genes were compared with our RNA‐seq data and the result showed gene expression of *FGFR2, NR4A1, PIK3R1, FGF18*, and *BCL2L11* were significantly upregulated in the CO group (Figure 4D).

### 3.4 PI3K/AKT signalling pathway contributes to the development of CO by regulating apoptosis

**FIGURE 5:**
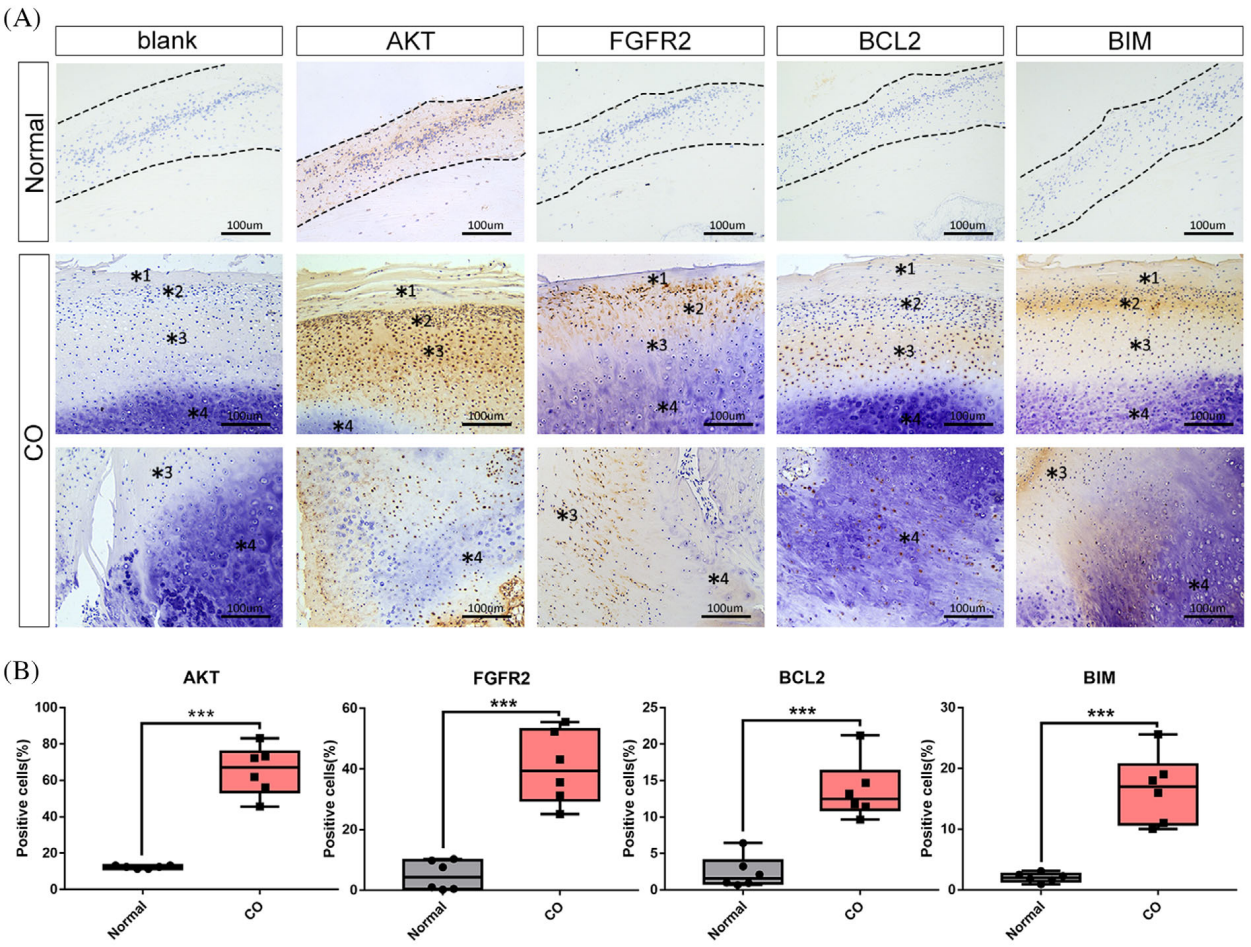
Immunohistochemical staining shows high levels of expression for AKT, FGFR2, BCL2 and BIM in CO. (A) Representative staining of AKT, FGFR2, BCL2 and BIM in normal condyle tissue and CO tissue. (B) AKT, FGFR2, BCL and BIM are all expressed at significantly higher levels in CO tissue than in normal tissue. Images are at 10 × 20 (=200) magnification (***p ˂ 0.001). *1, superficial zone; *2, proliferating zone; *3, pre‐hypertrophic zone; *4, hypertrophic zone.

It was evident that there are significant expression differences of hub genes in PI3K/AKT signalling in the CO group. We therefore investigated protein expression in CO tissue by immunohistochemical analysis and scored the staining intensity. As shown in Figure 5A,B, strong AKT, FGFR2, BCL2, and BCL2‐like protein 11 (BIM) staining was more frequent in CO tissue than in the normal condyle. And staining was positive in different zones. Abundant AKT is expressed in the superficial and proliferative zones. FGFR2 is highly expressed in the superficial and hypertrophic zones, whereas BCL2 is highly expressed in the polymorphic zone but BIM is mainly expressed in the proliferative zone.

**FIGURE 6:**
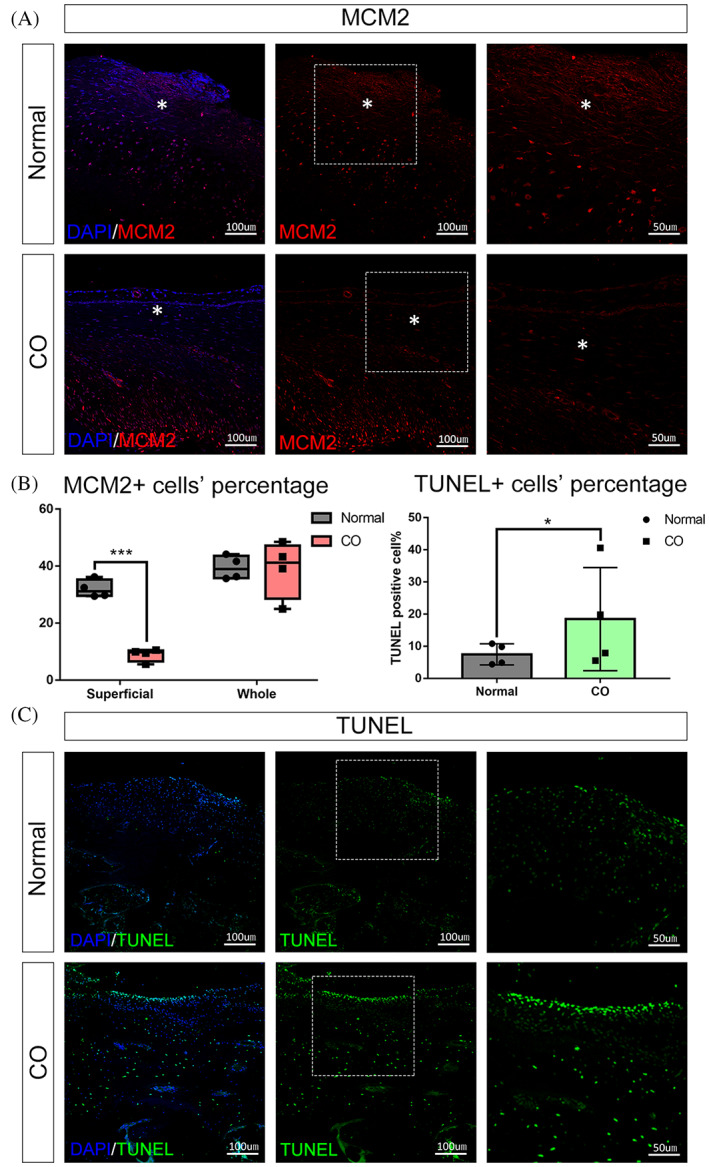
CO tissue has lower percentage of cell propagation at superficial zone and more cell apoptosis in the whole layer. (A),(B) (left): Immunohistochemical staining of MCM2 in normal condyle and CO tissue shows a higher percentage in the superficial zone but similar percentage of cell propagation in the whole layer of CO tissue in comparison to normal tissue. (B) (right) and (C) cell apoptosis in tissue detected using TUNEL labeling. CO tissue showed more cell apoptosis in the whole layer. Images are at 10 × 10 (=100) and 10 × 20 (=200) magnification (*p ˂ 0.05, ***p ˂ 0.001). *, superficial zone.

Next, TUNEL assay and IHC for MCM2 were used to measure apoptosis and proliferation in CO and normal tissue due to the clear role of BCL2 family proteins in regulating apoptosis. More MCM2 positive cells were found in the superficial zone of the normal condyle even though there was no significant difference between CO and normal tissue in the whole layer. (Figure 6A,B). The result of a TUNEL assay showed that CO tissue had a higher percentage of TUNEL positive cells than normal tissue (Figure 6B,C). Therefore, AKT mediates the level of BCL2 family proteins and increases cell apoptosis in CO.

**FIGURE 7:**
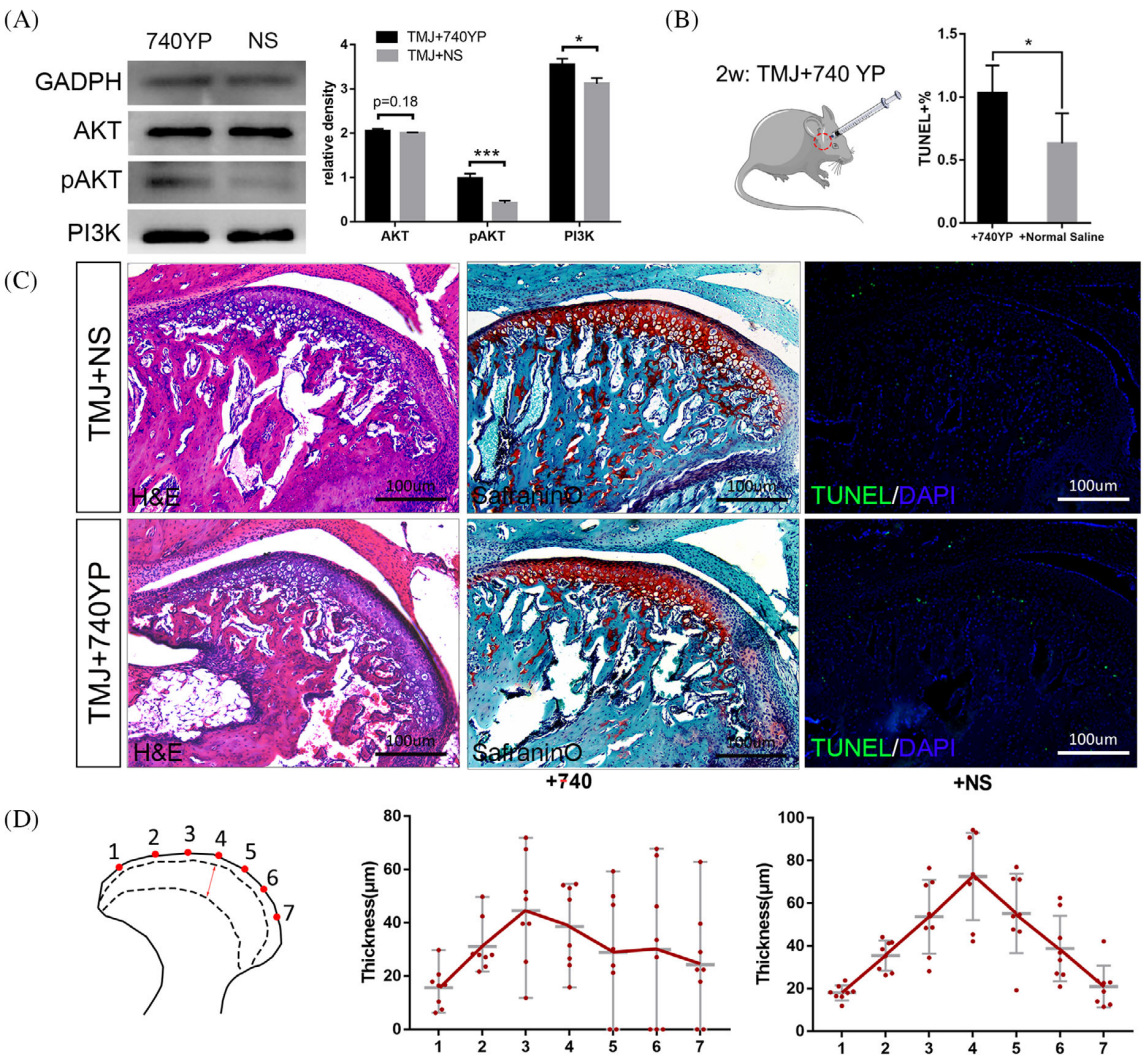
TMJ injected with PI3K activator showed histological disorder and a higher apoptosis ratio. (A) The expression of pAKT and PI3K significantly increased when 30 μM 740Y‐P was added into rat FCSCs for 24 h. (B),(C) The histological structure is disorganized in condyle cartilage in 740Y‐P group. The percentage of TUNEL positive cells in 740 Y‐P group is also higher than in the NS group. (D) The hypertrophic cartilage distribution of 740 Y‐P group is disordered compared with NS group. Images are at 10 × 10 (=100) magnification (*p ˂ 0.05, ***p ˂ 0.001). NS, normal saline, TMJ, temporomandibular joint.

We confirmed the influence of PI3K/AKT signalling on the condyle in vivo using C57/B6 mice by injecting PI3K activator 740 Y‐P into TMJ. We had previously confirmed the activation function of 740 Y‐P on cells using Western Blotting to demonstrate that PI3K/AKT signalling was activated in vitro (Figure 7A). In vivo, after injection on daily basis for 4 weeks, we observed obvious histological disorder in condylar cartilage in comparison to the saline‐injected control group. A smooth transition from superficial zone to hypertrophic zone could not be seen in activator injection group (Figure 7C). From the analytic result of hypertrophic cartilage distribution, we can conclude that the distribution of hypertrophic zone of 740 Y‐P injection group is out of order compared with saline injection group (Figure 7D). Meanwhile, the TUNEL assay shows that when the PI3K/AKT signalling pathway was activated, the ratio of apoptotic cells in the condyle became significantly higher (Figure 7B,C).

All the results indicate that abnormally activated PI3K/AKT signalling in CO is potential to lead to functional changes of hFCSCs by increased cell apoptosis, which results in TMJ cartilage lesion in CO patients and animals.

## 4 DISCUSSION

Significant progress has been made in the quest to understand the cell biology of osteochondroma, but there are several avenues of opportunity for further exploration. According to previous studies, loss‐of‐function mutations in exotoxin 1 (EXT1) and exotoxin 2 (EXT2) genes have been identified as the causative agents in HME.^16^, ^17^, ^18^, ^19^ However, Qin et al did not find that the EXT gene was the virulence gene for CO using gene sequencing analysis of 12 samples.^20^ CO did not develop as part of multiple osteochondromas, so its pathogenesis is different than HME. The significance of growth plate regulation to the pathogenesis of osteochondroma has been demonstrated,^21^ so we postulated that the superficial layer of the condyle might regulate the developmental process of CO.

We used hFCSCs, a stem cell population residing in the superficial layer of condylar cartilage, to determine the cell‐of‐origin for CO. In this study, we isolated hFCSCs from CO tissue for the first time. The fibrous superficial layer of CO becomes thicker than in normal condyle, but the proportion of hFCSCs in CO decreases as shown by FACS. The characteristics of the stem cell population is also changed under pathological conditions. Compared to normal hFCSCs, hFCSCs from CO have weaker propagation capacity and a higher cell apoptosis percentage. Both types of hFCSCs can be induced to become chondrocytes in vitro but the chondrogenic pattern of hFCSCs from CO is significantly different from normal. Thus the chondrogenic characteristics of this stem cell population has been altered in the tumour microenvironment.

However, identifying which gene or signalling pathway that regulates the function of hFCSCs during the pathological process of CO still requires validation and comprehensive study. However, our RNA‐seq analysis identified the PI3K/AKT signalling pathway has a significant role and is a likely candidate. Several studies have reported that the PI3K/AKT signalling pathway exhibits pleiotropic functions in chondrogenesis. PI3K/AKT signalling is required for the proliferation and the production of sulfated GAG in human articular chondrocytes.^22^, ^23^, ^24^ In the early chondrogenic differentiation stage, PI3K/AKT preserves the chondrogenic phenotype and inhibits the transition to terminal differentiation.^25^, ^26^ But it drives terminal differentiation once entering into the hypertrophic differentiation stage.^27^ The gene changes observed in hFCSCs from CO, together with previous studies, suggest that the chondrogenic homeostasis in normal condylar cartilage development might be affected via the activation of the PI3K/AKT signalling pathway.

Decades of research have confirmed that elevated PI3K/AKT signalling can contribute to tumorigenesis and it is a hallmark of human cancer. AKT phosphorylation serves as a surrogate readout of PI3K activation in the PI3K/AKT signalling pathway.^28^ This tight coupling of PI3K and AKT regulates a wide range of target proteins that control cell survival, growth, and other processes, including the proapoptotic BCL2 family proteins.^29^ The BCL2 family proteins are subdivided into three groups based on different functions or functional domains, such as the anti‐apoptotic protein BCL2 and the pro‐apoptotic protein BIM.^30^ Our RNA‐Seq analysis of hFCSCs from CO showed that PI3K/AKT signalling pathway activation leads to functional changes correlated to cell survival. Furthermore, altered expression levels of proteins involved in PI3K/AKT signalling in CO tissues verified the activation of the pathway. Upstream signalling factors combine with ligands such as FGFR2 to activate PI3K and AKT by secondary messengers PIP_3_ and lead to the higher expression of BCL2 and BIM. The altered expression level of BCL2 and BIM correlate to the cell apoptotic homeostasis and combine to contribute to abnormal cell survival in the different layers of human CO tissue.

Osteochondroma that originates in different bones still has similar histological characteristics, exhibiting the formation of cartilaginous matrix capped by a dense fibrous layer.^31^ Previous studies have reported several signalling pathways that contribute to osteochondroma. For example, the involvement of BMP2 signalling in the growth of the cartilage cap in osteochondroma has been demonstrated.^31^ Moreover, Judith et.al^32^ reported variation in expression of FGF2, FGFR2, FGFR3, PTHrP, and PTHrP‐R in osteochondroma samples from HME and non‐HME patient. However, only BCL2 expression was stronger in non‐HME cases compared with HME cases. This demonstrates a protein expression pattern similar to our results and the pathological difference between HME and non‐HME. In addition, Wnt/β‐catenin signalling and Ihh signalling have been associated with regulation of tumour size or fate.^33^ We report for the first time that PI3K/AKT signalling is related to CO by regulating apoptosis. Even though disorder in the mouse condyle was observed histologically after PI3K activation, severe and obvious hyperplasia was not seen during a one‐month period. CO has chronic development, and a longer observation time or a more effective method such as transgenic mice will be required for further study.

## 5 CONCLUSION

We detected significant differences in the cellular characteristics and in the activation of PI3K/AKT signalling between hFCSCs from CO and normal condyles. This suggests a new hypothesis regarding the cell‐of‐origin for human CO and another possible target for treatment.

## AUTHOR CONTRIBUTIONS

Conceptualization, Ruiye Bi and Songsong Zhu; Methodology, Qing Yin, Haohan Li and Ruiye Bi; Formal analysis, Qing Yin, Haohan Li, and Peiran Li; Investigation, Qing Yin; Writing‐Original Draft, Qing Yin, Qianli Li, and Ruiyu Wang; Writing‐Review & Editing, Ruiye Bi and Songsong Zhu; Visualization, Qing Yin, Haohan Li and Ruiye Bi; Supervision, Songsong Zhu; Project Administration, Songsong Zhu; Funding Acquisition, Ruiye Bi and Songsong Zhu.

## FUNDING INFORMATION

This work was funded by grants from the National Natural Science Foundation of China (No. 81771097, 82071139, 81801003) and ‘From Zero to One’ Innovative Research Program of Sichuan University (No. 2022SCUH0022).

## CONFLICT OF INTEREST

The authors declare that there is no conflict of interest.

## Supporting information


**Appendix S1.** Supporting Information.

## Data Availability

The data that support the findings of this study are available from the corresponding author upon reasonable request.
