# Supplementary material for: Regulatory role of human fibrocartilage stem cells in condyle osteochondroma
Source: Cell Prolif. 2022 Sep 26;56(1):e13342. doi: 10.1111/cpr.13342 (PMC9816926; doi:10.1111/cpr.13342)
Supplement: Supplementary file 1 — Appendix S1. Supporting Information. [file CPR-56-e13342-s001.docx]

**Regulatory role of human fibrocartilage stem cells**

**in condyle osteochondroma**

Qing Yin^1^, Ruiye Bi^1^, Haohan Li^1^, Qianli Li^1^, Peiran Li^1^, Ruiyu Wang^1^, Songsong Zhu^*1^

**Experimental procedures**

***Fluorescence-activated cell sorting (FACS)***

The first passage hFCSCs were removed from the dishes by trypsin. After resuspension, the cells were blocked in PBS+40% FBS for 15 minutes at room temperature. A total of 10^6^ cells were incubated with PE anti-human CD90 antibody (328109, Biolegend, USA), PerCP/Cyanine5.5 anti-human CD34 antibody (34361, Biolegend, USA), FITC anti-human CD73 antibody (344015, Biolegend, USA) and PE-Cy7 anti-human CD105 antibody (25-1057-42, eBioscience, USA) in staining buffer (PBS+1% BSA) on ice for 30 minutes. The cells were resuspended in FACS flow buffer (PBS+1% BSA+25mM Hepes PH7+5mM EDTA) and sorted on a BD Moflo XDP cell sorting system. The positive gate was established by blank control. The sorted cells were collected in sterilized centrifuge tubes with complete culture medium and incubated in culture dishes at 37°C and 5% CO_2_.

***EdU labeling and TUNEL assay***

A Click-iT® EdU cell proliferation kit (C10338, Invitrogen, USA) was used for measuring proliferation capacity of hFCSCs from normal condyle and CO. Cells were seeded in a 24-well plate at 2*10^4^ cells/well. Half of the medium was replaced by 20μM EdU to make the final solution 10mM. Cells were then fixed with 4% paraformaldehyde after two-hour incubation with EdU and were reacted with Click-iT staining cocktail in the dark. The nuclei were counterstained with DAPI (H-1200, Vector laboratories, USA). Positive cells were observed and counted under fluorescence microscope (DMi8, Leica, Germany). Percentage of EdU-positive cells was calculated by number of red-fluorescent (Alexa Fluor® 555-stained) cells/ number of DAPI-stained cells.

TUNEL staining was performed using the In Situ Cell Death Detection Kit (11684809910, Roche, Switzerland). Fixation and staining process were according to manufacturer’s instructions.

***Chondrogenic inducing differentiation***

HFCSCs were seeded in V-bottom 96 well plates (Corning, USA) at a density of 2.5*10^5^ cells/well in DMEM high glucose medium (SH30243, Hyclone, USA) supplemented with 1*10^-7^ M dexamethasone (D4902, Sigma-Aldrich), 1 mM sodium pyruvate (P4562, Sigma-Aldrich), 50μg/ml L-ascorbic-2-phosphate (A4403, Sigma-Aldrich), 40μg/ml L-proline (P5607, Sigma-Aldrich), 1% insulin, transferrin, selenium (ITS, 12521, Sigma-Aldrich), and 10 ng/ml TGF-β3 (100-36E-10, PeproTech). Cells were aggregated by centrifugation and incubated for 24-48h to create pellets. The pellets were incubated at 37°C and 5% CO_2_ in the dark for one and two weeks.

***Immunocytochemistry***

HFCSCs were seeded in 12-well plate with cell slides at a density of 2.5*10^4^ cells/well and cultured overnight. The cells were fixed with 4% paraformaldehyde for 10 minutes at room temperature, permeated with 0.1% Triton X-100 ([9002-93-1](https://www.sigmaaldrich.com/catalog/search?term=9002-93-1&interface=CAS%20No.&N=0&mode=partialmax&lang=en&region=HK&focus=product), Sigma-Aldrich, USA) for 20 minutes and then blocked with 10% goat serum(16210064, Gibco, USA) for one hour at room temperature. Subsequently, the cells were incubated with antibodies against SOX9 (ab3697, Abcam, USA) and MMP13 (JF0893, Huabio, China) diluted 1:100 in PBS overnight at 4℃. The cells were washed three times with PBST and incubated with Alexa Fluor 488 (A11008, Invitrogen, USA) and phalloidin (P1951, Sigma-Aldrich, USA) for 40 minutes at room temperature. Mountant with DAPI was used for nuclear staining. Immunostaining was visualized by fluorescence microscopy (FV1000, Olympus, Japan).

***Immunohistochemistry staining***

Sections of samples were deparaffinized and rehydrated. Antigen retrieval was performed by incubation for 20 minutes at 92.5-98.5℃ with PH 8.0 1*EDTA (C1034, Solarbio, China). After blocking with 10% goat serum for one hour, sections were incubated with antibodies against MMP13, SOX9 and ACAN (MA3-16888, Invitrogen, USA) or MCM2 (3619S, Cell Signaling Technology) and were incubated with Alexa Fluor 488 or Alexa Fluor 568 for 30 minutes at room temperature. Finally, mountant with DAPI was stained and immunostaining was imaged. For immunostaining of FGFR2 (23328S, Cell Signaling Technology), AKT (9272S, Cell Signaling Technology), BCL2 (ET1702-53, Huabio, China) or BIM (ET1608-14, Huabio, China), next biotin/HRP was labeled by common SP kit (SP-9000, ZSGB Bio, China).

***Quantitative PCR***

Total RNA from pellets was extracted from five pellets of each replicate using TRIzol reagent ([10296010](https://www.thermofisher.com/order/catalog/product/10296010), Invitrogen, USA). Total RNA was reverse-transcribed to cDNA using the PrimeScript RT reagent kit (RR047, Takara, Japan). Quantitative real-time PCR was performed on the StepOnePlus Real-Time PCR System (Quant Studio 3, Applied Biosystems, USA) using [TB Green™ Premix Ex Taq II](http://www.baidu.com/link?url=AbtZ_LYHjNZCmXlLdT2ZV0v_MccTqqvqqBhPve-mmUyIKlyYBjmFYQPP-7eLy65Y0lhBKr53DI4EiphJcHLIN_) (RR820, Takara, Japan). The sequences of the forward and reverse primers are listed in Table S2. Data were normalized against the corresponding levels of human GAPDH.

***Western blot***

The lysates from pellets were extracted from 5 pellets of each replicate. Protein from each group of pellets was extracted by NE-PER Nuclear and Cytoplasmic Extraction Reagents (78833, Thermo Fisher, USA) in liquid nitrogen tissuelyser. Protein samples were heated in loading buffer containing 5% 2-mercaptoethanol at 95°C for five minutes. Then denatured samples were electrophoresed in gels made by Bio-Rad 10% TGX Stain-Free FastCast Kit (1610183, Bio-Rad, USA), and the resolved proteins were transferred to Immobilon-PVDF membrane (ISEQ00010, Millipore, USA). Membranes were blocked with 5% BSA+ Tris-buffered saline with Tween (TBST), washed in TBST and incubated with rabbit anti-Collagen II (1:1000, ab34712, Abcam, USA) antibodies, mouse anti-GAPDH (1:5000, ab9482, Abcam, USA) and rabbit anti-MMP13 (1:1000, JF0893, Huabio, China). Goat anti-rabbit IgG-HRP (1:5000, ab6712, Abcam, USA) was used as secondary antibody. Immunoreactivity was detected with ECL Substrate (1705060, Bio-Rad, USA) and photographed on Bio-Rad ChemiDoc MP Imaging System. Band densities were calculated using Image Lab software.

**Figure S1**

**
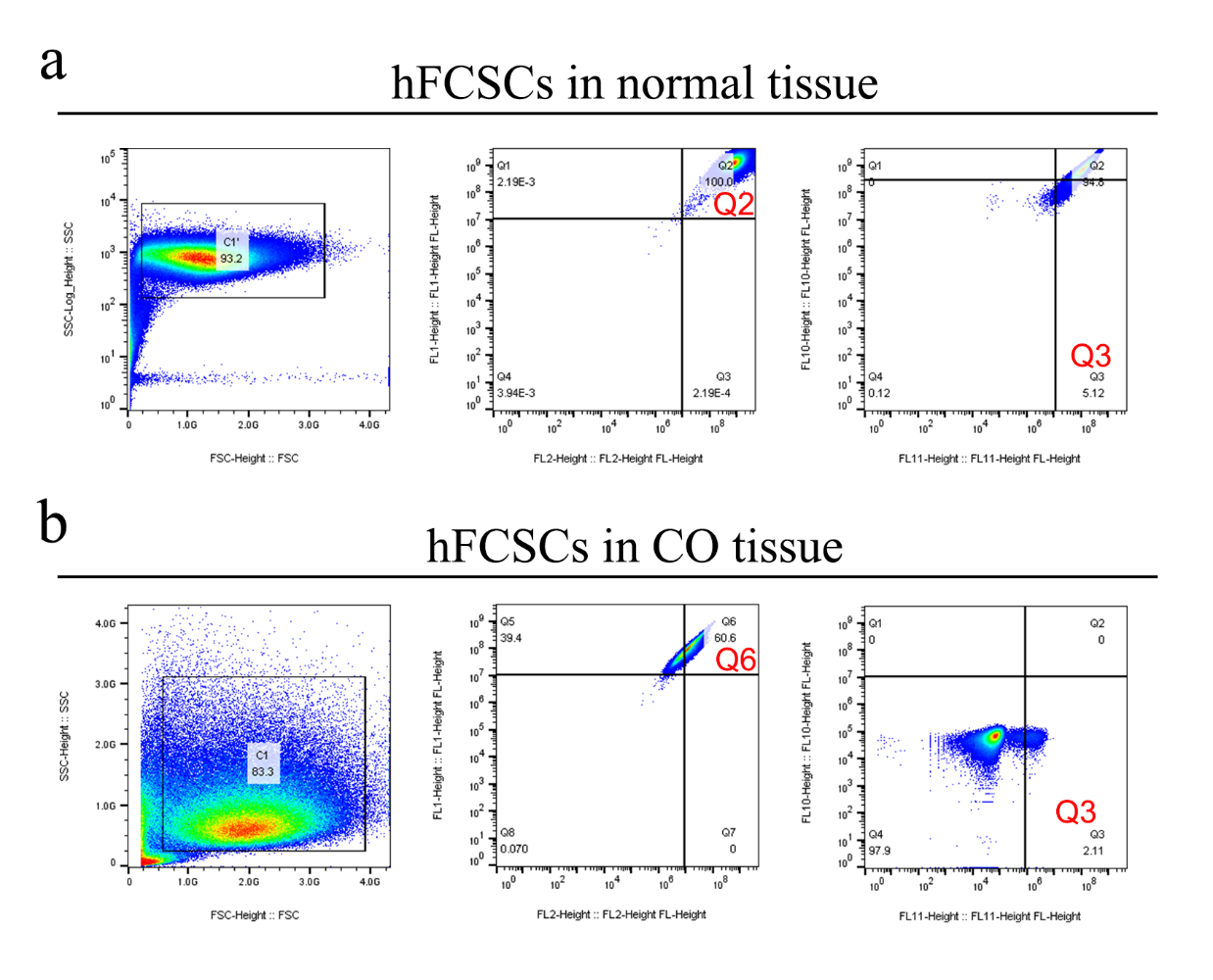
**

Figure S1. **Purification of hFCSCs from CO and normal condyle.** All samples were incubated with PE anti-human CD90 antibody, PerCP/Cyanine5.5 anti-human CD34 antibody, FITC anti-human CD73 antibody and PE-Cy7 anti-human CD105 antibody for 30 minutes. After excluding adhesions and cell debris, cells were purified with the standard of CD34-/CD73+/CD90+/CD105+. Q2 and Q6 include cells with CD90+/CD73+ labeling. Q3 includes cells with CD34-/CD73+/CD90+/CD105+ labeling.

**Table S1**

**Different genetic expression in hFCSCs between normal and CO**

| Gene name | Fold change | Regulation |
| --- | --- | --- |
| BCL2L11 | 2.64 | up |
| BDNF | 0.15 | down |
| CCNE2 | 0.37 | down |
| CHRM2 | 0.06 | down |
| COL4A5 | 0.11 | down |
| COMP | 5.70 | up |
| CSF1 | 2.03 | up |
| EREG | 0.10 | down |
| F2R | 0.15 | down |
| FGF18 | 3.22 | up |
| FGF2 | 2.55 | up |
| FGF5 | 0.21 | down |
| FGF7 | 3.68 | up |
| FGF9 | 15.91 | up |
| FGFR2 | 2.39 | up |
| FLT1 | 0.02 | down |
| GHR | 2.07 | up |
| IL7R | 0.37 | down |
| ITGA2 | 0.26 | down |
| ITGA3 | 0.41 | down |
| KITLG | 0.35 | down |
| KIT | 0.22 | down |
| LAMA1 | 0.28 | down |
| LAMA5 | 2.18 | up |
| LAMC2 | 3.28 | up |
| NGF | 2.66 | up |
| NR4A1 | 2.53 | up |
| NTRK2 | 2.90 | up |
| PIK3R1 | 2.16 | up |
| PPP2R2B | 0.16 | down |
| PRKAA2 | 0.42 | down |
| RELN | 0.10 | down |
| SPP1 | 0.20 | down |
| TGFA | 0.07 | down |
| THBS3 | 2.02 | up |
| TLR4 | 3.40 | up |
| TNXB | 4.33 | up |

**Table S2**

**Primers table**

| **Name** | **Sequence** |
| --- | --- |
| GAPDH F | GGGTGTGAACCATGAGAAGT |
| GAPDH R | AGTCCTTCCACGATACCAAAGT |
| SOX9 F | TAAAGG CAACTCGTACCCAAA T |
| SOX9 R | GTCCAGTTTCTCGTTGATTTC G |
| MMP13 F | CACTCCTTAGGTCTTGACCACTC |
| MMP13 R | AGAGAGACTGGATCCCTTGTACA |
| COL2A1 F | CTCAAGTCCCTCAACAACCAGAT |
| COL2A1 R | AGTAGTCTCCACTCTTCCACTCA |
| COL10A1 F | CAACAGCATTATGACCCAAGGAC |
| COL10A1 R | TGCCATTCTTATACAGGCCTACC |
| FGF2 F | TGTAGAAGATGTGACGCCGC |
| FGF2 R | GTTCACGGATGGGTGTCTCC |
| FGF5 F | CAGTAGCGCTATGTCTTCCTCTT |
| FGF5 R | ATCTTTGGCTTGATAGGGCTAGG |
| FGF7 F | GATACTGACATGGATCCTGCCAA |
| FGF7 R | TTGCCATAGGAAGAAAGTGGGC |
| FGF9 F | ATGCGGTACCGTTTGGGAAT |
| FGF9 R | TGATCCAAGTCCGTGACTGC |
| FGFR2 F | AGCACTCGGGGATAAATAGTTCC |
| FGFR2 R | CTGGTTGGCCTGCCCTATATAAT |
| NR4A1 F | ATGTACAGCAGTTCTACGACCTG |
| NR4A1 R | CCTCCAGCTTGAGGTAGAAGATG |
| PPP2R2B F | CTCCATATCTGTCAACAGCGACT |
| PPP2R2B R | GTTGTAGGAGCCTGTCATGATGA |
| PIK3R1 F | CGAGCCCTATAACTTGTACAGCT |
| PIK3R1 R | TACTGGGTAGGCTAGTGTGACAT |
| FGF18 F | TCCTAGTGGAGACAGACACCTTC |
| FGF18 R | CTTCTCGATGAACACACACTCCT |
| AKT1 F | CAGGATGTGGACCAACGTGA |
| AKT1 R | AAGGTGCGTTCGATGACAGT |
| BAX F | AACCATCATGGGCTGGACATT |
| BAX R | CCACAAAGATGGTCACGGTCT |
| MTORC1 F | CTCAGTGGTACAGGCACACAT |
| MTORC1 R | AGGATCAACAAGGCTCCATGG |
| FOXO1 F | ACCTGTACAAGTGCCTCTGC |
| FOXO1 R | GCGCTCAATGAACATGCCAT |
| BCL2L11 F | GCCTGGTCTGCAGTTTGTTG |
| BCL2L11 R | ATCAAGTGGTGGTAGTGGCG |
| BCL2 F | GGATAACGGAGGCTGGGATG |
| BCL2 R | GGCCAAACTGAGCAGAGTCT |

**Table S3**

**Patients’ information**

| **Surgery date** | **Gender** | **Age** | **Diagnosis** |
| --- | --- | --- | --- |
| 20170818 | female | 47 | Right CO |
| 20170614 | female | 28 | Right CO |
| 20180528 | male | 25 | Right CO |
| 20180531 | male | 27 | Right CO |
| 20181126 | male | 34 | Left CO |
| 20190222 | male | 40 | Right CO |
| 20190409 | female | 34 | Left CO |
| 20200617 | female | 48 | Right CO |
| 20170819 | male | 10 | Left condylar bone fracture |
| 20170825 | male | 17 | Right condylar bone fracture |
| 20170726 | female | 22 | Bilateral condylar bone fracture |
| 20171024 | male | 10 | Left condylar bone fracture |
| 20180620 | male | 35 | Bilateral condylar bone fracture |
